# Signal Transduction Pathways in Plants for Resistance against Pathogens

**DOI:** 10.3390/ijms20092335

**Published:** 2019-05-11

**Authors:** Jian-Zhong Liu, Hon-Ming Lam

**Affiliations:** 1 College of Chemistry and Life, Zhejiang Normal University, Jinhua 321004, China; 2 School of Life Sciences and Center for Soybean Research of the State Key Laboratory of Agrobiotechnology, The Chinese University of Hong Kong, Shatin, Hong Kong, China

Plants are constantly exposed to a diverse group of pathogens and have evolved sophisticated immune systems to combat pathogen attacks [1,2,3]. Upon pathogen attack, plasma membrane-localized pattern recognition receptors (PRRs), including receptor-like kinases (RLKs) or receptor-like proteins (RLPs), recognize pathogen-associated molecular patterns (PAMPs) to activate PAMP-triggered immunity (PTI) [2]. In response, some pathogens can deliver effector proteins into host cells to suppress the PTI. As a counter measure, the pathogen effectors can be recognized by the intracellular nucleotide-binding leucine-rich repeat (NLR) immune receptors either directly or indirectly, which in turn can activate effector-triggered immunity (ETI) [2,3]. Both PTI and ETI share significant overlapping downstream defense signaling events, but ETI is much faster-acting and mounts a more effective response than PTI [2,4]. The downstream signal transduction events may include NAPDH oxidase-dependent reactive oxygen species (ROS) production, activation of Ca^2+^, mitogen-activated protein kinase (MAPK) and trimeric G-protein signaling pathways, induction of salicylic acid (SA) biosynthesis, *Pathogenesis-Related* (*PR*) genes, callose deposition and biosynthesis of phytoalexins, as well as transcriptome reprogramming [2,5,6,7,8,9]. The defense signaling pathways are interconnected to form a defense network, in which numerous transcription factors, biochemical pathways, and phytohormones are involved. 

This Special Issue consists of nine research articles and seven review articles. One feature of this Special Issue is that the “-omics” approach is widely used among the research articles, representing a new trend in research. However, because of the dynamic nature of the “-omics” experiments, how to extract the biologically meaningful information from big data is still a challenge. The review articles in this Special Issue summarize and integrate the latest advances in many different aspects of plant defense signaling, which will serve as valuable resources for those who are interested in plant immunity. 

Bacterial pathogens gain entry into the leaf apoplasts through open stomatal pores [10]. Plants have evolved a mechanism to close stomata upon sensing PAMPs, known as stomatal defense [10,11]. It has been shown previously that a small GTPase, Nucleolar GTP-Binding Protein 12 (NOG1-2), acts as a positive regulator of stomatal closure in response to both abiotic and biotic stresses [12]. In this Special Issue, Lee et al. (2018) took a step further and showed that NOG1-2 participates in stomatal closure through interacting with JAZ9 and multiple other JAZ proteins [13]. NOG1-2 inhibits the interaction between JAZ9 and COI1, and both NOG1-2 and JAZ9 co-regulate a set of the core genes involved in jasmonic acid (JA) and abscisic acid (ABA) signaling pathways, implying that NOG1-2 is involved in the coordination and integration of JA and ABA pathways to modulate stomatal closure in response to biotic and abiotic stresses. 

Leaf chlorosis is the most common viral infection symptom, which has been assumed to be the result of virus-induced changes in chloroplast structure and components [14,15,16]. However, the molecular mechanism that underlies chlorosis as a result of viral infection is not fully understood. Zhao et al. (2018) identified 66 nuclear-encoded chloroplast proteins that failed to be transported to the target plastids during *Rice stripe virus* (RSV) infection in *N. benthamiana* using an elegantly designed experiment in combination with a label-free nano LC-MS/MS. Among these 66 proteins, three nuclear-encoded chloroplast proteins are involved in the chloroplast-targeting biological process itself [17]. Thus the authors proposed that RSV infection blocks the normal localization and functions of these three proteins, resulting in the mis-targeting of many chloroplast-targeted proteins subsequently. As a consequence, both the structure and function of the chloroplasts are severely impaired. This is one of the successful examples showing the power of the “-omics” approach in understanding the molecular mechanism underlying a specific phenomenon or phenotype. 

Root restriction (RR) is a commonly used cultivation practice to improve the nutritional values of grape berries, by restricting the root volume and shoot growth [18,19,20]. Leng et al., (2018) investigated the effects of RR treatment on the accumulation levels of major phytohormones as well as on the transcriptome during berry development and ripening of an early-ripening seedless grape using Ultra Performance Liquid Chromatography-High Resolution Mass Spectrometry (UPLC-HRMS) and RNA sequencing (RNAseq), respectively [21]. Their results showed that RR treatment increased the levels of stress-related phytohormones but decreased the levels of phytohormones involved in growth and development. Most of the RR-responsive genes are involved in the phytohormone biosynthesis and signal transduction pathways. These results provided partial explanations for the RR-induced improvement of berry quality and the acceleration of berry ripening.

Satellite RNAs (satRNAs) depend on their cognate helper viruses for their replication in host cells. satRNAs usually decrease helper virus titers and attenuate symptom severity [22,23]. However, the molecular mechanism underpinning this reduced virulence remains an enigma. Wrzesinska et al. (2018) performed proteomic and phosphoproteomic analyses on plants infected with Peanut stunt virus (PSV) either alone or co-infected with its satellite RNA (satRNA) in *N. benthamiana* and found that PSV-only infection caused a strong reduction in the number of phosphorylated proteins and the phosphorylation levels, whereas PSV+satRNA co-infection resulted in significant reductions in overall protein levels in *N. benthamiana* plants [24]. The differentially phosphorylated proteins were mostly associated with photosynthesis, carbon metabolism, and RNA processing. Although these results are helpful for understanding the molecular mechanisms of the reduced virulence resulting from the PSV+satRNA co-infection, much work is still needed to identify the specific targets of the phosphorylation and the protein targets that can account for the reduced virulence observed in co-infected plants.

Zhang et al (2018) identified a nuclear-localized Squamosa-promoter binding protein (SBP) from pepper (*Capsicum annuum*), CaSBP12, as a negative regulator of defense response [25]. Silencing *CaSBP12* enhanced the resistance against *Phytophthora capsici*, whereas over-expressing *CaSBP12* had the opposite effect. Using a gain-of-function approach, Huang et al. (2018) showed that *Stilbene Synthase 36* from Chinese wild *Vitis quinquangularis* (*VqSTS36*) plays a positive role in the resistance against the biotrophic fungal pathogen, powdery mildew, but a negative role in the resistance against the necrotrophic fungal pathogen, *Botrytis cinereal* [26]. In addition, *VqSTS36* also positively regulates salt and drought resistance.

SD20 is one of few cultivars of potatoes that are resistant to a super race isolate of *Phytophthora infestans* (*Pi*), CN152. Yang et al (2018) performed an RNAseq analysis to systematically identify the SD20-specific late blight-responsive genes by comparing the gene expression profiles between SD20 and a susceptible cultivar upon CN152 infection [27]. They identified the differentially expressed genes (DEGs) enriched in the GO-terms that have been previously shown to be involved in immune responses and hypersensitive responses. Tariq et al (2018) performed a comparative transcriptomic profiling of two near-isogenic lines (NILs) of rice, CBB23 (harboring the *R* gene *Xa23*) and JG30 (without *Xa23*), before and after infection with the *Xanthomonas oryzae* pv *oryzae* strain PXO99A, to identify genes associated with the disease resistance [28]. The vast majority of DEGs identified between CBB23 and JG30 are enriched in phenylpropanoid biosynthesis, followed by phytohormone pathways, flavonoid biosynthesis, and glycolysis/gluconeogenesis. Heat-stable activity factor (HSAF) is produced in many microbes with a broad-spectrum antifungal activity. *Alternaria alternata* (Fries) Keissler is a lethal fungal pathogen that causes leaf black spot disease in pear. He et al. (2018) showed that HSAF inhibited the mycelial growth of *A. alternata* in a dose-dependent manner [29]. Transcriptomic analysis revealed that the HSAF treatment disrupted multiple signaling networks and essential cellular metabolisms and breached metabolic networks and induced thickening of the cell wall and apoptosis in *A. alternata*. This study can potentially help to develop novel bio-fungicides.

Plants are constantly exposed to a variety of abiotic and biotic stresses simultaneously. It is critical to understand how plants recognize and respond to these distinct stimuli and integrate the various responsive signals to combat these stresses. Ku et al. (2018) thoroughly reviewed how the crosstalks among different signaling pathways coordinate and balance the defense signaling in response to biotic and abiotic stresses [30]. Understanding the integrated actions of these different signaling pathways enables the rational design of strategies to cope with multiple stresses using transgenic approaches. Ali et al. (2018) extensively reviewed the molecular interactions between plants and nematodes, with a major focus on the mechanism by which the nematodes use for parasitizing their hosts [31]. The authors described how the nematode feeding sites (NFSs) are established by CLAVATA3-RELATED peptide (CLE) mimicry and other mechanisms. Nematodes produce and deliver a repertoire of effector proteins into plant cells to suppress or evade defense responses (both PTI and ETI), shift metabolic pathways, and change developmental programs in the hosts for their own benefit, through reprogramming the transcriptomes and modulating phytohormone pathways of their hosts [32,33]. Plants have evolved strategies to deter herbivores and yet attract insect pollinators and other beneficial insects [34]. Meanwhile, insects have evolved strategies to overcome the plant defense barriers in order to feed on plants [34,35]. In this Special Issue, Li et al. reviewed how plants and insects use miRNAs to regulate their biological processes, respectively, and co-opt each other’s miRNA systems for their own purposes [36]. However, the insect- and plant-derived miRNAs and their respective targets have not been identified yet and the impact of these miRNAs on insect physiology remains controversial. Unlike other plant-pathogen interactions, in which the NLR-type *R* genes play the major roles, the resistance of rice against *X. oryzae* is mostly associated with the transcription activator-like effector (TALEs) [37]. The resistance against *X. oryzae* by rice uses two main strategies: activating the host’s innate immunity upon perception of the pathogen effectors, and abolishing the host’s susceptibility through a loss of interaction with effectors [38]. Ji et al. (2018) further categorized these mechanisms into five different routes and described these routes individually in detail [39]. 

Accumulated evidence indicates that BRs are actively involved in plant–environment interactions. Yu et al. (2018) highlighted the recent advances in the understanding of brassinosteroid (BR) functions in modulating plant interactions with different pathogenic microbes, with a particular focus on how BR signaling primes the plant innate immunity pathways and achieves a trade-off between growth and immunity [40]. The authors summarized almost all aspects of BR involvements in regulating plant-microbe interactions. As master regulators of numerous downstream target genes, the transcription factors (TFs) involved in defense responses are also subjected to regulations. Ng et al (2018) extensively reviewed the regulation of TFs at the epigenetic, transcriptional/post-transcriptional, and translational/post-translational levels [41]. The authors also proposed the potential applications of TFs in crop improvement. 

Three major wheat fungal pathogens belonging to different trophic types use distinct strategies to infect wheat. Duba et al (2018) reviewed the current status of disease resistance research in wheat, ranging from the mapping and identification of resistance genes to the unraveling of mechanisms of resistance at the anatomical, morphological, and molecular levels [42]. 

We hope that this Special Issue will provide our readers with a framework for understanding the defense signaling pathways and provide insights into new research directions in this field. We thank all authors for their contributions and thank the reviewers for their critical assessment of these articles. 

## References

[1] Dangl J.L., Jones J.D. (2001). Plant pathogens and integrated defence responses to infection. Nature.

[2] Jones J.D., Dangl J.L. (2006). The plant immune system. Nature.

[3] Chisholm S.T., Coaker G., Day B., Staskawicz B.J. (2006). Host-microbe interactions: shaping the evolution of the plant immune response. Cell.

[4] Peng Y., van Wersch R., Zhang Y. (2018). Convergent and Divergent Signaling in PAMP-Triggered Immunity and Effector-Triggered Immunity. Plant Microbe Interact..

[5] Meng X., Zhang S. (2013). MAPK cascades in plant disease resistance signaling. Annu. Rev. Phytopathol..

[6] Kadota Y., Shirasu K., Zipfel C. (2015). Regulation of the NADPH Oxidase RBOHD during Plant Immunity. Plant Cell Physiol..

[7] Yuan P., Tanaka K., Du L., Poovaiah B.W. (2018). Calcium Signaling in Plant Autoimmunity: A Guard Model for AtSR1/CAMTA3-Mediated Immune Response. Mol. Plant.

[8] Zhang Y., Li X. (2019). Salicylic acid: biosynthesis, perception, and contributions to plant immunity. Curr. Opin. Plant Biol..

[9] Zhong C.L., Zhang C., Liu J.Z. (2018). Hetero-trimeric G protein signaling in plant immunity. J. Exp. Bot..

[10] Melotto M., Underwood W., Koczan J., Nomura K., He S.Y. (2006). Plant stomata function in innate immunity against bacterial invasion. Cell.

[11] Melotto M., Zhang L., Oblessuc P.R., He S.Y. (2017). Stomatal Defense a Decade Later. Plant Physiol..

[12] Lee S., Senthil-Kumar M., Kang M., Rojas C.M., Tang Y., Oh S., Choudhury S.R., Lee H.-K., Ishiga Y., Allen R.D. (2017). The small GTPase, nucleolar GTP-binding protein 1 (NOG1), has a novel role in plant innate immunity. Sci. Rep..

[13] Lee S., Rojas C.M., Oh S., Kang M., Choudhury S.R., Lee H.-K., Allen R.D., Pandey S., Mysore K.S. (2018). Nucleolar GTP-Binding Protein 1-2 (NOG1-2) Interacts with jasmonate-ZIMDomain Protein 9 (JAZ9) to regulate stomatal aperture during plant immunity. Int. J. Mol. Sci..

[14] Bhattacharyya D., Gnanasekaran P., Kumar R.K., Kushwaha N.K., Sharma V.K., Yusuf M.A., Chakraborty S. (2015). A geminivirus betasatellite damages the structural and functional integrity of chloroplasts leading to symptom formation and inhibition of photosynthesis. J. Exp. Bot..

[15] Li Y., Cui H., Cui X., Wang A. (2016). The altered photosynthetic machinery during compatible virus infection. Curr. Opin. Virol..

[16] Zhao J., Zhang X., Hong Y., Liu Y. (2016). Chloroplast in plant-virus interaction. Front. Microbiol..

[17] Zhao J., Xu J., Chen B., Cui W., Zhou Z., Song X., Chen Z., Zheng H., Lin L., Peng J. (2019). Characterization of proteins involved in chloroplast targeting disturbed by rice stripe virus by vovel protoplast–chloroplast proteomics. Int. J. Mol. Sci..

[18] Xie Z.S., Li B., Forney C.F., Xu W.P., Wang S.P. (2009). Changes in sugar content and relative enzyme activity in grape berry in response to root restriction. Sci. Hortic..

[19] Wang B., He J., Yang B., Yu X., Li J., Zhang C., Xu W., Bai X., Cao X., Wang S. (2013). Root restriction affected anthocyanin composition and up-regulated the transcription of their biosynthetic genes during berry development in ‘summer black’ grape. Acta Physiol. Plant.

[20] Wang B., He J., Duan C., Yu X., Zhu L., Xie Z., Zhang C., Xu W., Wang S. (2012). Root restriction affects anthocyanin accumulation and composition in berry skin of ‘kyoho’ grape (vitis vinifera L. × vitis labrusca L.) during ripening. Sci. Hortic..

[21] Leng F., Cao J., Wang S., Jiang L., Li X., Sun C. (2018). Transcriptomic analyses of root restriction effects on phytohormone content and signal transduction during grape berry development and ripening. Int. J. Mol. Sci..

[22] Simon A.E., Roossinck M.J., Havelda Z. (2004). Plant virus satellite and defective interfering RNAs: New paradigms for a new century. Annu. Rev. Phytopathol..

[23] Liao Q., Zhu L., Du Z., Zeng R., Feng J., Chen J. (2007). Satellite RNA-mediated reduction of cucumber mosaic virus genomic RNAs accumulation in Nicotiana tabacum. Acta Biochim. Biophys. Sin..

[24] Wrzesińska B., Dai Vu L., Gevaert K., De Smet I., Obrępalska-Stęplowska A. (2018). Peanut stunt virus and its satellite RNA trigger changes in phosphorylation in *N. benthamiana* infected plants at the early stage of the infection. Int. J. Mol. Sci..

[25] Zhang H.-X., Ali M., Feng X.-H., Jin J.-H., Huang L.-J., Khan A., Lv J.-G., Gao S.-Y., Luo D.-X., Gong Z.-H. (2019). A novel transcription factor *CaSBP12* gene negatively regulates the defense response against *Phytophthora capsica* in pepper (*Capsicum annuum* L.). Int. J. Mol. Sci..

[26] Huang L., Yin X., Sun X., Yang J., Rahman M.Z., Chen Z., Wang X. (2018). Expression of a grape *VqSTS36*-increased resistance to powdery mildew and ssmotic stress in Arabidopsis but enhanced susceptibility to *Botrytis cinerea* in Arabidopsis and tomato. Int. J. Mol. Sci..

[27] Yang X., Guo X., Yang Y., Ye P., Xiong X., Liu J., Dong D., Li G. (2018). Gene profiling in late blight resistance in potato genotype SD20. Int. J. Mol. Sci..

[28] Tariq R., Wang C., Qin T., Xu F., Tang Y., Gao Y., Ji Z., Zhao K. (2018). Comparative transcriptome profiling of rice near-isogenic line carrying *Xa23* under infection of *Xanthomonas oryzae* pv. oryzae. Int. J. Mol. Sci..

[29] He F., Li B., Ai G., Kange A.M., Zhao Y., Zhang X., Jia Y., Dou D., Liu F., Cao H. (2018). Transcriptomics analysis of the Chinese pear pathotype of *Alternaria alternate* gives insights into novel mechanisms of HSAF antifungal activities. Int. J. Mol. Sci..

[30] Ku Y.-S., Sintaha M., Cheung M.-Y., Lam H.-M. (2018). Plant hormone signaling crosstalks between biotic and abiotic stress responses. Int. J. Mol. Sci..

[31] Ali M.A., Anjam M.S., Nawaz M.A., Lam H.-M., Chung G. (2018). Signal transduction in plant–nematode interactions. Int. J. Mol. Sci..

[32] Hewezi T., Baum T. (2012). Manipulation of plant cells by cyst and root-knot nematode effectors. Mol. Plant Microbe Interact..

[33] Hewezi T. (2015). Cellular Signaling pathways and posttranslational modifications mediated by nematode effector proteins. Plant Physiol..

[34] Williamson V.M., Gleason C.A. (2003). Plant-nematode interactions. Curr. Opin. Plant Biol..

[35] Gheysen G., Mitchum M.G. (2011). How nematodes manipulate plant development pathways for infection. Curr. Opin. Plant Biol..

[36] Li C., Wong A.Y.P., Wang S., Jia Q., Chuang W.-P., Bendena W.G., Tobe S.S., Yang S.H., Chung G., Chan T.-F., Lam H.-M., Bede J.C., Hui J.H.L. (2018). MiRNA-mediated interactions in and between plants and insects. Int. J. Mol. Sci..

[37] Liu W.D., Wang G.L. (2016). Plant innate immunity in rice: A defense against pathogen infection. Natl. Sci. Rev..

[38] Kourelis J., van der Hoorn R.A.L. (2018). Defended to the nines: 25 years of resistance gene cloning identifies nine mechanisms for R protein function. Plant Cell.

[39] Ji Z., Wang C., Zhao K. (2018). Rice routes of countering *Xanthomonas oryzae*. Int. J. Mol. Sci..

[40] Yu M.-H., Zhao Z.-Z., He J.-X. (2018). Brassinosteroid signaling in plant–microbe interactions. Int. J. Mol. Sci..

[41] Ng D.-K., Abeysinghe J.K., Kamali M. (2018). Regulating the regulators: The control of transcription factors in plant defense signaling. Int. J. Mol. Sci..

[42] Duba A., Goriewa-Duba K., Wachowska U. (2018). A Review of the Interactions between Wheat and wheat pathogens: *Zymoseptoria tritici*, *Fusarium* spp. and *Parastagonospora nodorum*. Int. J. Mol. Sci..

